# Nitrogen and chlorophyll status determination in durum wheat as influenced by fertilization and soil management: Preliminary results

**DOI:** 10.1371/journal.pone.0225126

**Published:** 2019-11-14

**Authors:** Marco Fiorentini, Stefano Zenobi, Elisabetta Giorgini, Danilo Basili, Carla Conti, Chiara Pro, Elga Monaci, Roberto Orsini

**Affiliations:** 1 Department of Agricultural, Food and Environmental Sciences (D3A), Section of Agronomy and Crop Science, Marche Polytechnic University, Ancona, Italy; 2 Department of Life and Environmental Sciences (DISVA), Section of General and Inorganic Chemistry, Marche Polytechnic University, Ancona, Italy; University of Vigo, SPAIN

## Abstract

Handheld chlorophyll meters as Soil Plant Analysis Development (SPAD) have proven to be useful tools for rapid, no-destructive assessment of chlorophyll and nitrogen status in various crops. This method is used to diagnose the need of nitrogen fertilization to improve the efficiency of the agricultural system and to minimize nitrogen losses and deficiency. The objective of this study is to evaluate the effect of repeated conservative agriculture practices on the SPAD readings, leaves chlorophyll concentration and Nitrogen Nutrition Index (NNI) relationships in durum wheat under Mediterranean conditions. The experimental site is a part of a long-term-experiment established in 1994 and is still on-going where three tillage managements and three nitrogen fertilizer treatments were repeated in the same plots every year. We observed a linear relationship between the SPAD readings performed in the central and distal portion of the leaf (R^2^ = 0.96). In fertilized durum wheat, we found all positive exponential relationships between SPAD readings, chlorophyll leaves concentration (R^2^ = 0.85) and NNI (R^2^ = 0.89). In the unfertilized treatment, the SPAD has a good attitude to estimate leaves chlorophyll concentration (R^2^ = 0.74) and NNI (R^2^ = 0.77) only in crop grow a soil with relative high content of soil organic matter and nitrogen availability, as observed in the no tilled plots. The results show that the SPAD can be used for a correct assessment of chlorophyll and nitrogen status in durum wheat but also to evaluate indirectly the content of soil organic matter and nitrogen availability during different growth stages of the crop cycle.

## Introduction

The essential goal of world agriculture is to provide sufficient amount of food to satisfy the nutritional demand of the current population. In the next 31 years, a growth of 2.6 billion people is estimated [1]. There is a growing demand for food that needs to be filled [2] while reducing production costs and pollution.

The reduction of costs and pollution in cultivation systems inevitably goes by the adoption of conservative agriculture (CA) practices concerning soil management [3] and a better management of production inputs. In fact, the reduced tillage and even the no-tillage bring benefits to the environment in terms of reduction of soil erosion, leaching of nitrates, reduction in the use of agricultural machinery as well as a lower emission of greenhouse gases and fuel costs [4]. Furthermore, the low soil disturbance, with the addition of crop residues, increases the levels of humidity and nutrients in the horizons of soil explored from the roots, and reduces the mineralization rate of the organic matter [5].

These issues have been studied in the Mediterranean because winter cereals are the dominant crops [6]. In the Mediterranean area, crop production can be improved with the adoption of CA techniques [5] and with the application of the right dose of nitrogen (N), through the site-specific application of fertilizers [7].

N deficiency leads to low shoot biomass and reduces yield, while excessive N causes disease and a range of environmental issues [8]. Nitrogen use efficiency is a central issue and goal of applied research in agricultural systems to assess the N uptake by crops and can be used to apply N fertilizer avoiding pollution from mineral N input [9–11]. Fertilizer applications should be based on precise estimates of crop N requirements [12].

The Nitrogen Nutrition Index (NNI) was developed in France for diagnosing plant N status for range of cereals and grasses, and to adjust N fertilization [13–15]. Recent studies on durum wheat [16] and rice [17–18] confirm important progress in the management of nitrogen fertilization during the growth stages. However, NNI determination requires destructive time-consuming measurements of plant N content and crop biomass. Widely used methods for determining crop nitrogen requirements require substantial time, the use of sophisticated laboratory equipment, and associated costs. Time required for sample collection and analyses may disallow timely producer response to crop nitrogen deficiencies [19].

To overcome these logistical and economic problems, it is necessary to calibrate the NNI with other methods [16] as various types of portable diagnostic tools have been developed for assessing the N status of a crop within the growing season, to provide farmers with decision support based on different measurements [20]. Plant-sap nitrate concentration, leaf chlorophyll content and crop transmittance and reflectance are the most commonly used indicators on which N fertilizer recommendations are based [21–22].

Since there is a close relationship between chlorophyll and nitrogen in the leaf tissue content [23], scientific studies are being directed towards chlorophyll meter, a no-destructive and low-time consuming method [24–25]. Many studies have found a relationship between chlorophyll meter readings and leaves chlorophyll concentration in several cereal crops [26–28]. Furthermore, a relationship between the Soil Plant Analysis Development (SPAD) readings and N crop status [19,25,28–29] has been found to establish robust N diagnostic models based on the optimal indicators and to assess their ranges of application [30].

However, the identification of appropriate levels of nitrogen fertilization based on the SPAD readings obtained with the chlorophyll meter is still under study. Furthermore, it has been shown that those relationships depend on cultivar, cultural practice and environmental factors [19,26,29,31–32]. To our knowledge there are not information that provide for a comparison between conventional vs conservative systems for the parameters listed above.

The aim of this study that represents the preliminary result of the research project "Setting of a precision farming robotic laboratory for cropping system sustainability and food safety and security" [33] is to evaluate the effect of CA practices on the SPAD readings, leaves chlorophyll concentration and NNI relationship in durum wheat under Mediterranean conditions for which no data are yet available.

## Materials and methods

### Experimental site

The experimental site is located at the “Pasquale Rosati” experimental farm of the Polytechnic University of Marche in Agugliano, Italy (43°32’N,13°22’E, at an altitude of 100 m above sea level and a slope gradient of 10%), on a silty-clay soil classified as Calcaric Gleyic Cambisols [34]. The climate of site is Mediterranean, the average rainfall is 838 mm (1998–2018 period) (Table 1). November is the rainiest month (93 mm) while July the driest month (36 mm). The average minimum temperature was 11.4°C while the mean maximum temperature was 20.0°C.

**Table 1:** Monthly precipitations and mean minimum (T min) and maximum (T max) air temperatures comparison in the experimental (November 2017 – October 2018) and long-term (November 1998–October 2018) period.

Months	November	December	January	February	March	April	May	June	July	August	September	October	Average or Total
**Rainfall (mm)**													
**2017–2018**	124	96	29	173	143	37	95	48	57	25	19	37	883
***Long-term***	*93*	*87*	*54*	*68*	*85*	*70*	*73*	*54*	*36*	*41*	*92*	*84*	*838*
**Min air T (°C)**													
**2017–2018**	7.9	4.1	5.2	2.0	5.7	11.8	14.9	17.7	20.5	20.8	16.8	12.2	11.6
***Long-term***	*8.7*	*4.4*	*3.2*	*3.8*	*6.6*	*9.7*	*13.7*	*17.8*	*20.2*	*20.2*	*16.1*	*12.9*	*11.4*
**Max air T (°C)**													
**2017–2018**	15.7	11.9	12.8	8.3	13.3	21.5	23.7	27.8	30.8	31.0	26.9	21.6	20.4
***Long-term***	*15.3*	*10.9*	*9.6*	*11.0*	*14.9*	*18.8*	*23.5*	*28.1*	*30.8*	*30.7*	*25.4*	*20.6*	*20.0*

During the experimental period (November 2017 –October 2018) we observed on average a similar trend without significant differences in terms of rainfall and temperature (max and min).

### Experimental design and crop management

The experimental site is a part of a long-term-experiment established in 1994 and is still on-going consisting on a rainfed 2 years rotation with durum wheat (Triticum turgidum L. var. durum, cv. Grazia, ISEA) in rotation with maize (Zea Mays L., DK440 hybrid Dekalb Monsanto, FAO Class 300) [35].

The crop rotation was duplicated in two adjacent fields to allow both crops to be present each year; in this paper the results observed in durum wheat in 2018 are presented. Within each field, three tillage (T, main plot, 1500 m^2^) and three nitrogen fertilizer (N, sub-plot, 500 m^2^) treatments were repeated in the same plots every year and arranged according to a split plot experimental design with two replicates.

The conventional tillage (CT), that is representative of the business as usual tillage practice in the study area, and the reduced tillage (RT) plots were ploughed along the maximum slope every year by mouldboard (with 2 plows) at the depth of 40 cm or a chisel at a depth of 25 cm respectively in autumn. The seedbed was prepared with double harrowing before the sowing date.

The no tillage (NT) soil was left undisturbed except for crop residues, weed chopping, and total herbicide spraying prior to direct seed drilling.

Soil properties in compared experimental plots are indicated in Table 2. Soil sampling was made with a Hand Huger (mod. Suelo HA-3) after the sowing, before the first N fertilization. From each subplot 3 samples were taken for a total of 54 soil samples analyzed.

**Table 2:** Soil properties (0–20 cm layer) (± dev.st.) in compared experimental plots.

Parameters	NT	RT	CT
**Sand (g kg^-1^)**	127 (±21) a	125 (±18) a	120 (±19) a
**Silt (g kg^-1^)**	410 (±30) a	388 (±11) a	397 (±19) a
**Clay (g kg^-1^)**	463 (±36) a	487 (±10) a	483 (±22) a
**Soil organic matter (g kg^-1^)**	25.5 (±5.9) a	17.2 (±1.0) b	12.6 (±0.9) b
**Total nitrogen (g kg^-1^)**	1.68 (±0.30) a	1.17 (±0.03) b	0.98 (±0.03) b

NT, no-tillage; RT, reduced tillage; CT, conventional tillage.

^a,b^ values having a common letter are not significantly different at P level = 5%.

The three nitrogen fertilizer treatments N0 = 0 kg N ha^-1^, N1 = 90 kg N ha^-1^ and N2 = 180 kg N ha^-1^ were distributed in two rates. The N1 treatment was compliant with the agro-environmental measures adopted within the rural development plans at local scale. The N2 treatments was the business as usual N rate in the study area at the start of the experiment. The N0 treatment was chosen as a control. Dates (dd/mm/yyyy) of all agronomic practices in the three soil managements are reported in Table 3.

**Table 3:** Agronomic management practices adopted during the experimental years 2017–2018.

Agro-technique	Soil managements	Date
**Ploughing (40 cm)**	CT	02/10/2017
**Chisel (25 cm)**	RT	04/10/2017
**Weed control**^**a**^	NT	14/11/2017
**Harrowing and seed bed preparation**	CT and RT	20/11/2017
**P fertilization**^**b**^	All	21/11/2017
**Sowing**^**c**^	All	21/11/2017
**Pest control**^**d**^	All	24/04/2018
**N fertilization**^**e**^	All	29/03/2018
	All	30/04/2018
**Harvest**	All	06/07/2018

CT, conventional tillage; RT, reduced tillage; NT, no-tillage;

^a^ Glyphosate dose: 2.25 kg ha^-1^ of active ingredient;

^b^ 70 kg P_2_O_5_ ha^-1^

^c^ Seed rate: 220 kg ha^-1^; Row spacing: 0.17 m;

^d^ Azoxystrobin dose: 0.16 l ha^-1^; Cyproconazole dose: 0.16 l ha^-1^

^e^ N source: urea (46%); 50% of N distribution for each date.

### Measurements

#### SPAD measurements

In order to avoid Soil Plant Analysis Development (SPAD) readings with strong variations, we have performed the sampling during a fully-sunny day. SPAD readings were made by using the chlorophyll meter SPAD Minolta 502 (Konica Minolta Sensing 2003, Osaka, Japan). The functioning of the SPAD Minolta 502 is based on production of light by two silicon photodiodes, with one sensitive to red light (650 nm; peak chlorophyll absorbance) and the other sensitive to infrared radiation (940 nm; non-chlorophyll absorbance). Electrical currents converted from light received by the silicon photodiodes are received by a microprocessor, which linearizes the signal and calculates a SPAD (unit less) value according to equation (Eq 1) [36]:

$$SPAD\;readings=A\times \left[\log\left(\frac{\text{Ior}}{Ir}\right)-\log\left(\frac{Iof}{if}\right)+B\right]$$ (1)

Where:

A = constant;

B = constant;

Ior = current from red detectors with sample in place;

Ir = current from red infrared detectors with sample in place;

Iof = currents from red detectors with no sample;

If = currents from infrared detectors with no sample.

We have randomly selected three test areas for each sub-plot. At each test areas we have chosen 10 full expanded and intact leaves on which SPAD readings were made. The readings were taken on the central (SPAD CP) and distal portion (SPAD DP) of the leaves, at the same time slot, around midday (11:00–13:00 a.m.). Moving from one sub-plot to the next one, we carefully cleaning the optical sensor with an alcohol solution and we performed the calibration activity as suggested by the SPAD Minolta 502 instruction manual. The 10 leaves for each test area, after the SPAD readings, were cut and sealed in a plastic bag, placed in a portable refrigerator and transferred to the laboratory where the chlorophyll was analyzed.

#### Leaves chlorophyll concentration

The concentration of the leaf chlorophyll was performed according with the Arnon’s method [37].

We cut the 10 fully expanded and intact leaves acquired from the previous sampling activities and we weight it (Eq 2) until we obtained about 0.1 g with a precision balance of α = 0.0001 g. The leaf pigments were extracted in 80% acetone and leaf was pulverized completely by using the homogenizer (Bio-Gen PRO200) to obtain a completely white foliar tissue. Before the analysis we centrifuged them for 5 minutes at 7 rpm to separate solid and liquid substance, and then we added more solution of 80% acetone until we reached a final volume of 25 ml.

The determination of leaves chlorophyll concentration is carried out by using a spectrophotometer (Varian Cary 50 Scan UV-visible spectrophotometric): the absorbance’s (A) were measured at 663 and 645 nm. Total chlorophyll contents of each sample were computed from the equations described below, using following equation (Eq 2) [37]:

mg total chlorophyll/g tissue=20,2×(A645)+8,02×(A663)×V1000×W (2)

Where:

A = absorbance at specific wavelengths

V = final volume of chlorophyll extract

W = fresh weigh of tissue extracted.

#### Nitrogen crop status determination

Total nitrogen was determined on the epigeal portion of fresh plants by automated combustion analysis Dumas method [38–39] in an oxygen-enriched atmosphere at a high temperature in order to ensure complete combustion of the whole sample. The fresh plants sampled were taken on three different test areas at each sub-plot in a 1 m long-row and oven-dried at 80°C for 48 h. We have ground to pass a 0.5 mm, and before we analyzed for total nitrogen, we weighed the dry biomass. Total nitrogen was determined by using EA 1110 LECO CHNS-0 analyzer (Leco Corporation, St. Joseph, MI).

Starting from the total nitrogen results, the Nitrogen Nutrition Index (NNI) was calculated by dividing actual nitrogen concentration (Dumas method) by the critical nitrogen concentration using the wheat dilution curve [14,19]. To calculate the NNI we used the following equations (Eqs 3 and 4):

$$NNI=\frac{\%N}{N_{C}}$$ (3)

$$N_{C}=a\times DM^{-b}$$ (4)

Where:

% N = actual N concentration

*N*_*c*_ = critical N concentration

a = 5.35

DM = dry matter (g)

b = 0.442

All described measurements were performed at tillering stage (Zadoks Scale, ZS22) [40] (before the 1^st^ N fertilization), stem elongation (ZS35) (between the N fertilizations) and anthesis (ZS60) (after the 2^nd^ N fertilization) to evaluate crop development in relation to the nitrogen fertilization. At crop maturity (ZS92) we measured the total biomass yield (g m^-2^) expressed on a dry matter content basis using plants sampled on three test areas at each sub-plot in a 1 m long-row and oven-dried at 105°C for 48 h. In addition, on the same samples, we determine the Harvest Index (H.I.) (Eq 5), using a laboratory thresher to separate the grain from the biomass.

$$H.I.\;\left(Harvest\;Index\right)=\frac{Grain\;yield\;\left(g\right)}{Total\;Biomass\;\left(g\right)}$$ (5)

### Statistical analysis

All statistical analysis was performed with the R Statistical software. Before to do any statistical analysis, we performed a normality test (Shapiro-Wilk W test) to evaluate the data distribution. When data were normally distributed a one-way ANOVA was applied followed by a Tukey post-hoc analysis. When data were not normally distributed the Kruskal-Wallis test was used followed by a post-hoc analysis Dunn (P level = 5%). Regression analysis (P level = 0.1%) was used to evaluate the relationship between SPAD readings, leaves chlorophyll concentration and NNI. The coefficient of determination (R^2^) and relative root mean square errors (RMSE) were used to evaluate the ability of the SPAD Minolta 502 to estimate the dependent variables.

## Results

### SPAD readings

**Fig 1:**
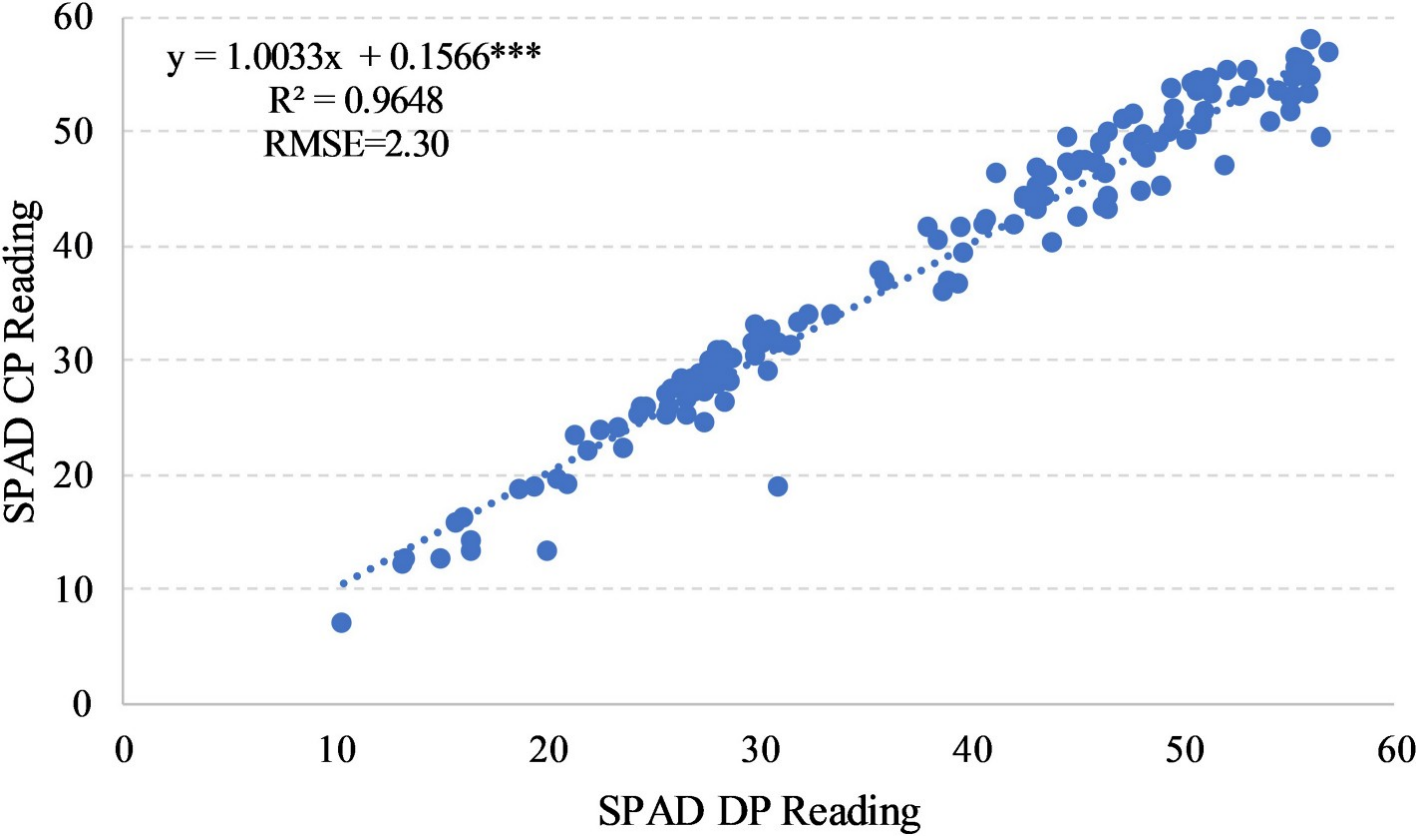
Relationship between SPAD readings observed on the central (SPAD CP) and distal portion (SPAD DP) in durum wheat during the season 2017–2018. ***: Significant at P level = 0.1%, RMSE, root mean square error.

The relationship between SPAD readings on the central (SPAD CP) and distal portion (SPAD DP) of the leaves that we observed is linear, with a coefficient of determination (R^2^) of 0.96 and a Root Mean Square Error (RMSE) of 2.30 (Fig 1).

On all data, the relationship between SPAD CP and SPAD DP was the following (Eq 6):

SPAD CP=1.0033 × SPAD DP+0.1566 (6)

As illustrated in Fig 1, the equation underlines that SPAD DP readings were generally lower than SPAD CP readings.

### SPAD readings and leaves chlorophyll concentration

**Fig 2:**
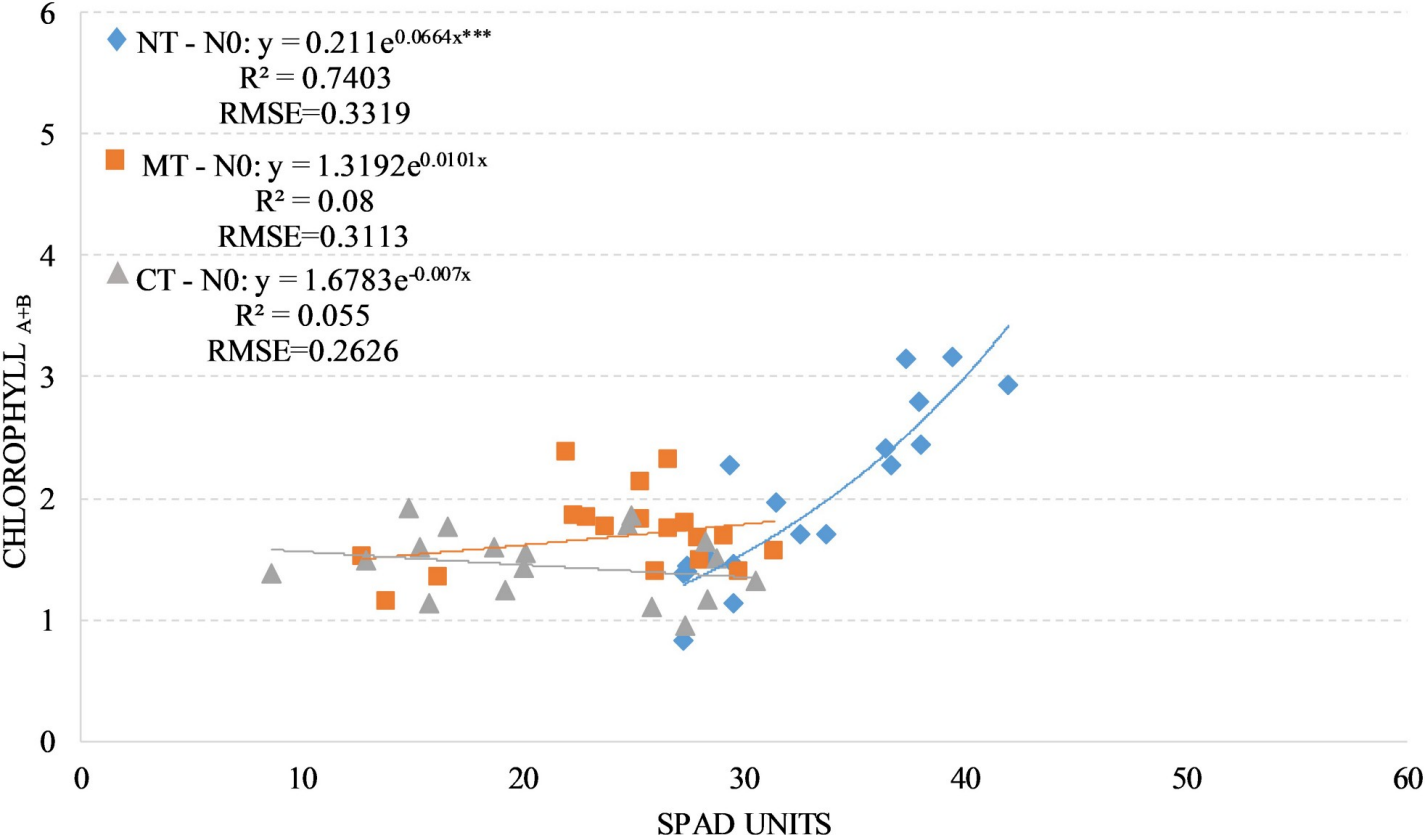
Relationship between leaves chlorophyll a+b concentration (mg/g) and SPAD readings in durum wheat during the season 2017–2018 under unfertilized treatment (N0, unfertilized treatment). NT, no tillage; RT, reduced tillage; CT, conventional tillage. ***: Significant at P level = 0.1%, RMSE, root mean square error.

We found a positive and significant exponential relationship between SPAD readings and leaves chlorophyll concentration for the factorial combinations NTxN0 (R^2^ = 0.74); no significant relationship where found for both factorial combination RTxN0 (R^2^ = 0.08) and CTxN0 (R^2^ = 0.05) with, in the latter case, a negative trend (CTxN0: y = 1.6783e^-0.007x^) (Fig 2).

By considering the SPAD readings and the leaves chlorophyll concentration, we found a significant difference between the factorial combinations NTxN0 compared to CTxN0. The factorial combination RTxN0 was not significantly different than CTxN0 for both destructive methods, while comparing RTxN0 and NTxN0 we have found only a significant difference on the SPAD readings (Table 4)

**Table 4:** SPAD readings (± dev.st.) and leaves chlorophyll _a+b_ concentration (mg/g) (± dev.st.) observed under unfertilized treatment in the different soil managements on the season 2017–2018.

Soil management	Nitrogen input	SPAD readings	Chorophyll _a+b_ concentration (mg/g)
**NT**	N0	32.89 (±4.88) a	2.00 (±0.71) a
**RT**	N0	24.30 (±5.29) b	1.72 (±0.33) ab
**CT**	N0	20.90 (±6.05) b	1.47 (±0.27) b
**Mean**		26.03 (±7.37)	1.73 (±0.52)

NT, no tillage; RT, reduced tillage; CT, conventional tillage; N0, unfertilized treatment.

^a,ab,b^ values having a common letter are not significantly different at P level = 5%.

**Fig 3:**
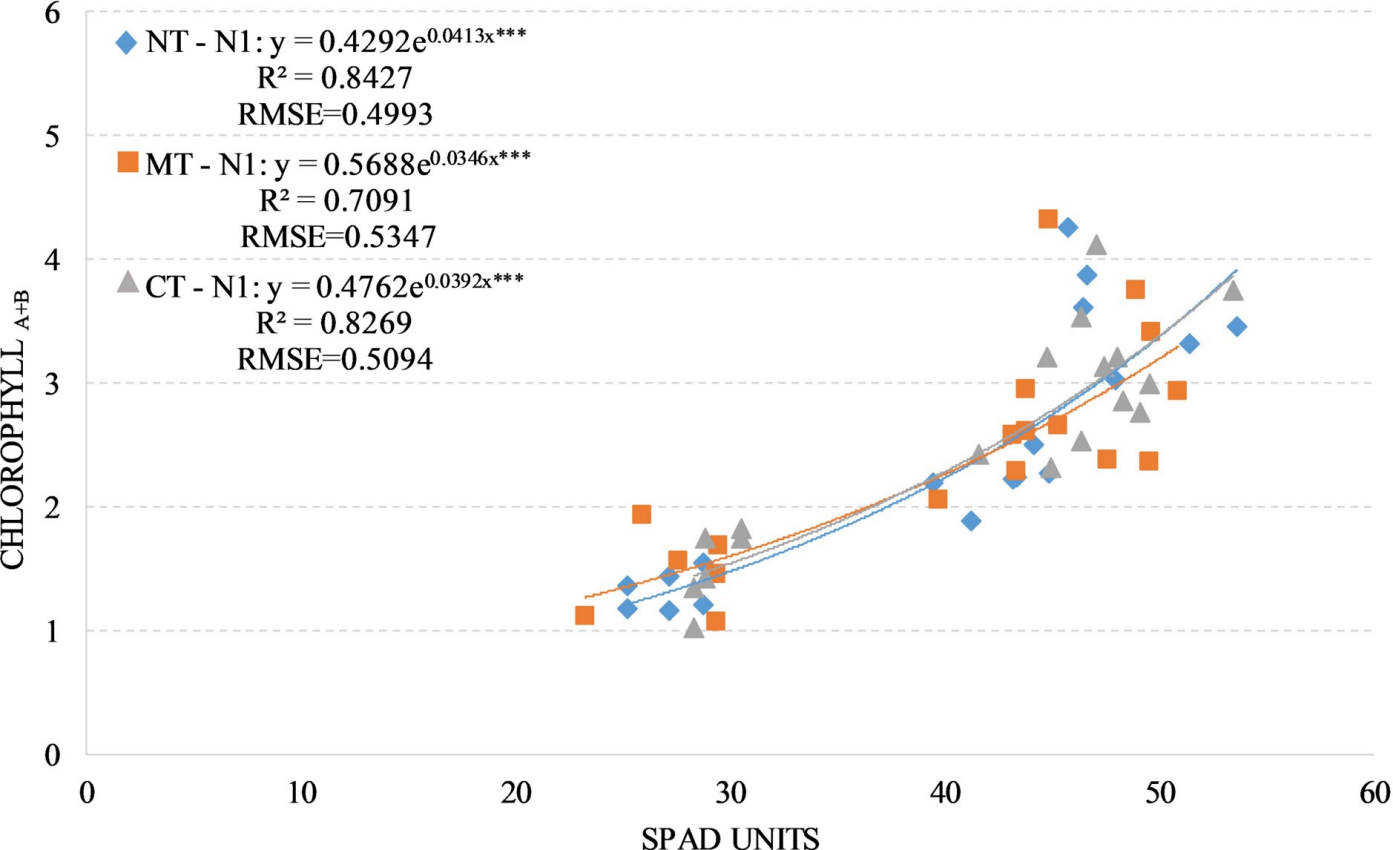
Relationship between leaves chlorophyll a+b concentration (mg/g) and SPAD readings in durum wheat during season 2017–2018 under N1 condition (N1, 90 kg N ha-1). NT, no tillage; RT, reduced tillage; CT, conventional tillage. ***: Significant at P level = 0.1%, RMSE, root mean square error.

**Fig 4:**
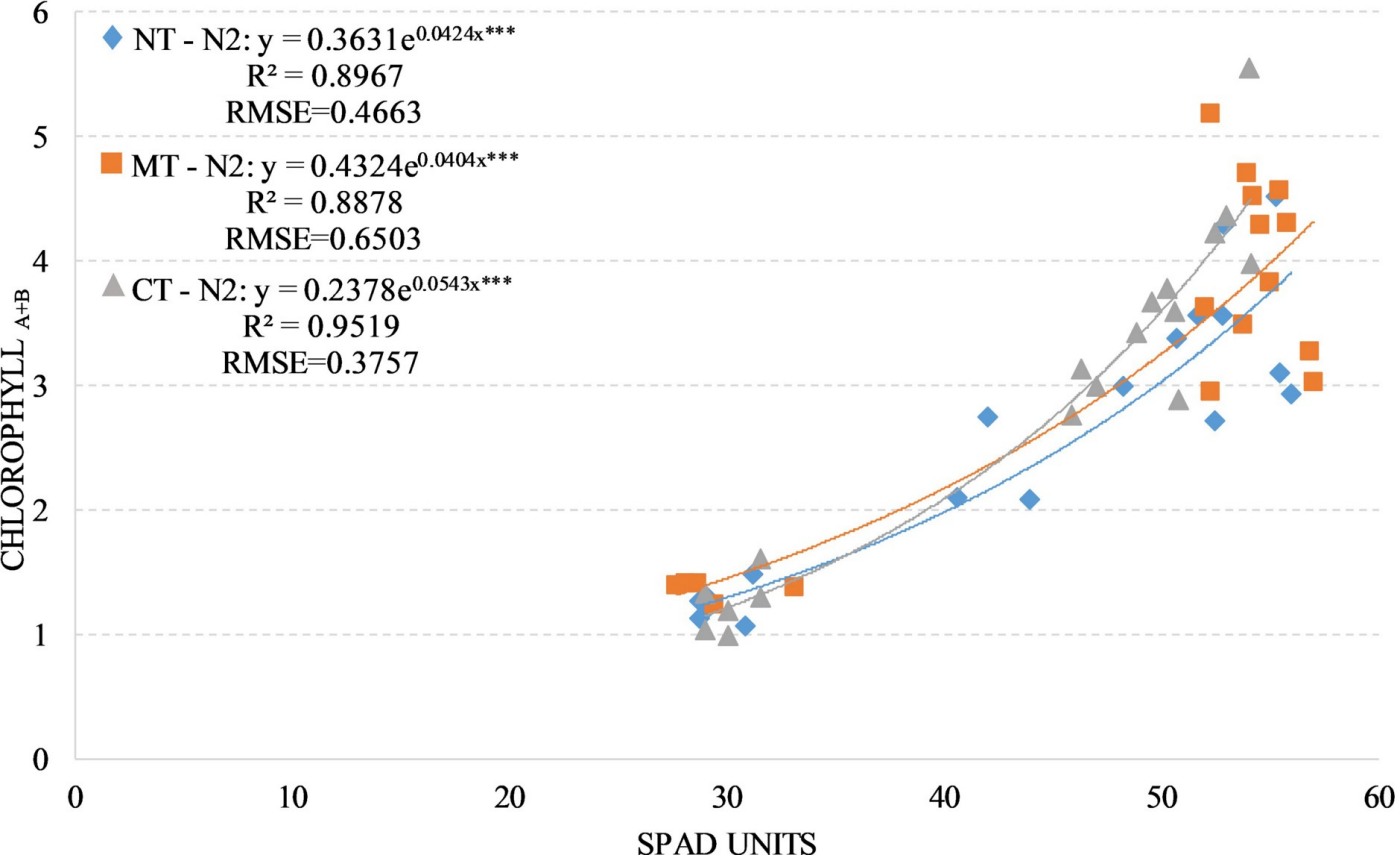
Relationship between leaves chlorophyll a+b concentration (mg/g) and SPAD readings in durum wheat during the season 2017–2018 under N2 condition (N2, 180 kg N ha-1). NT, no tillage; RT, reduced tillage; CT, conventional tillage. ***: Significant at P level = 0.1%, RMSE, root mean square error.

We observed positive and significant exponential relationships between the SPAD readings and leaves chlorophyll concentration under the two fertilized treatments (N1 and N2) regardless of soil management with an average R^2^ values of 0.85 and an average RSME of 0.50 approximately. The value of R^2^ change between the soil managements taking the two fertilized treatments individually (Figs 3 and 4).

By evaluating absolute values of SPAD readings and leaves chlorophyll concentration, we did not find a significant difference between the soil management under the two fertilized treatments (Table 5).

**Table 5:** SPAD readings (± dev.st.) and leaves chlorophyll _a+b_ concentration (mg/g) (± dev.st.) observed under N1 and N2 fertilized treatments in the different soil managements on the season 2017–2018.

Soil management	Nitrogen input	SPAD readings	Chorophyll _a+b_ concentration (mg/g)
**NT**	N1	39.45 (±9.62) a	2.38 (±1.00) a
**RT**	N1	39.75 (±9.42) a	2.40 (±0.88) a
**CT**	N1	41.23 (±9.06) a	2.56 (±0.89) a
**Mean**		40.15 (±9.23)	2.45 (±0.91)
**NT**	N2	43.33 (±10.85) a	2.53 (±1.11) a
**RT**	N2	46.02 (±12.36) a	3.12 (±1.39) a
**CT**	N2	43.58 (±9.99) a	2.89 (±1.35) a
**Mean**		44.31 (±10.97)	2.85 (±1.29)

NT, no tillage; RT, reduced tillage; CT, conventional tillage; N1, 90 kg N ha^-1^; N2, 180 kg N ha^-1^.

^a^ value having a common letter are not significantly different at P level = 5%.

### SPAD readings, Nitrogen Nutrition Index and total biomass yield

**Fig 5:**
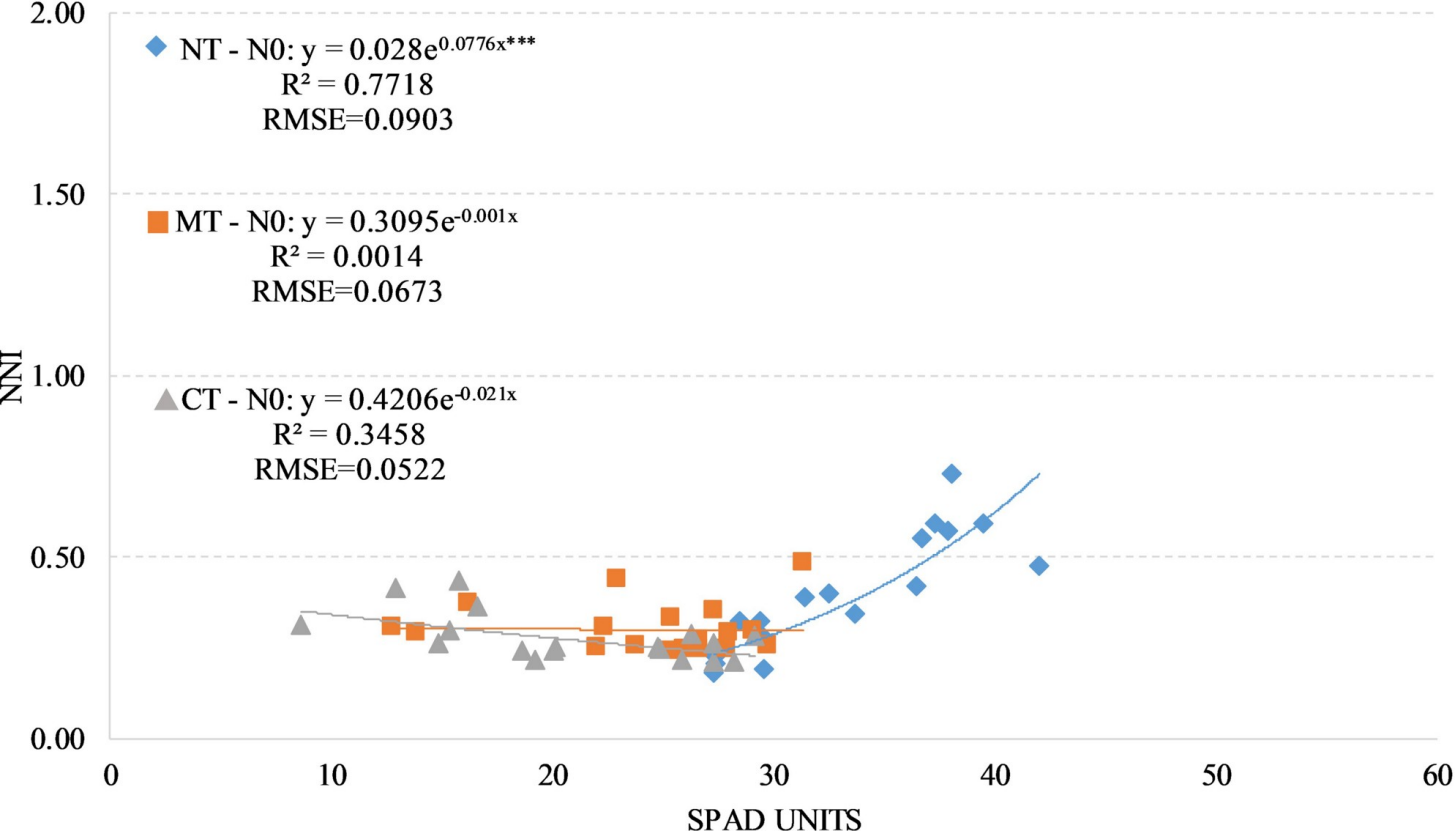
Relationship between Nitrogen Nutrition Index (NNI) and SPAD readings in durum wheat during the season 2017–2018 under unfertilized treatment (N0, unfertilized treatment). NT, no tillage; RT, reduced tillage; CT, conventional tillage. ***: Significant at P level = 0.1%, RMSE, root mean square error.

We observed a positive and significant exponential relationship between SPAD readings and NNI for the factorial combinations NTxN0 (R^2^ = 0.77); no significant relationship were found for both factorial combination RTxN0 (R^2^ = 0.01) and CTxN0 (R^2^ = 0.35) also highlighting a negative relationship (RTxN0: y = 0.3095e^-0.001x^; CTxN0; y = 0.4206e^-0.021x^) (Fig 5).

By analyzing the NNI values, we found a significant difference between the factorial combinations NTxN0 compared to CTxN0 (Table 6). The factorial combination RTxN0 was not significantly different from the factorial combination CTxN0 not from the NTxN0. The same dynamic was observed by analyzing the total biomass yield and the harvest index.

**Table 6:** Nitrogen Nutrition Index (NNI), total biomass yield and Harvest Index (HI) values observed (± dev.st.) under unfertilized treatment in the different soil managements on the season 2017–2018.

Soil management	Nitrogen input	NNI	Total biomass yield (g/m^2^)	HI
**NT**	N0	0.39 (±0.16) a	686.28 (±42.75) a	0.37 (±0.01) a
**RT**	N0	0.31 (±0.07) ab	452.91 (±64.60) b	0.30 (±0.01) b
**CT**	N0	0.28 (±0.07) b	421.00 (±72.98) b	0.31 (±0.01) b
**Mean**		0.32 (±0.12)	520.06 (±134.68)	0.32 (±0.03)

NT, no tillage; RT, reduced tillage; CT, conventional tillage; N0, unfertilized treatment.

^a,ab,b^ values having a common letter are not significantly different at P level = 5%.

**Fig 6:**
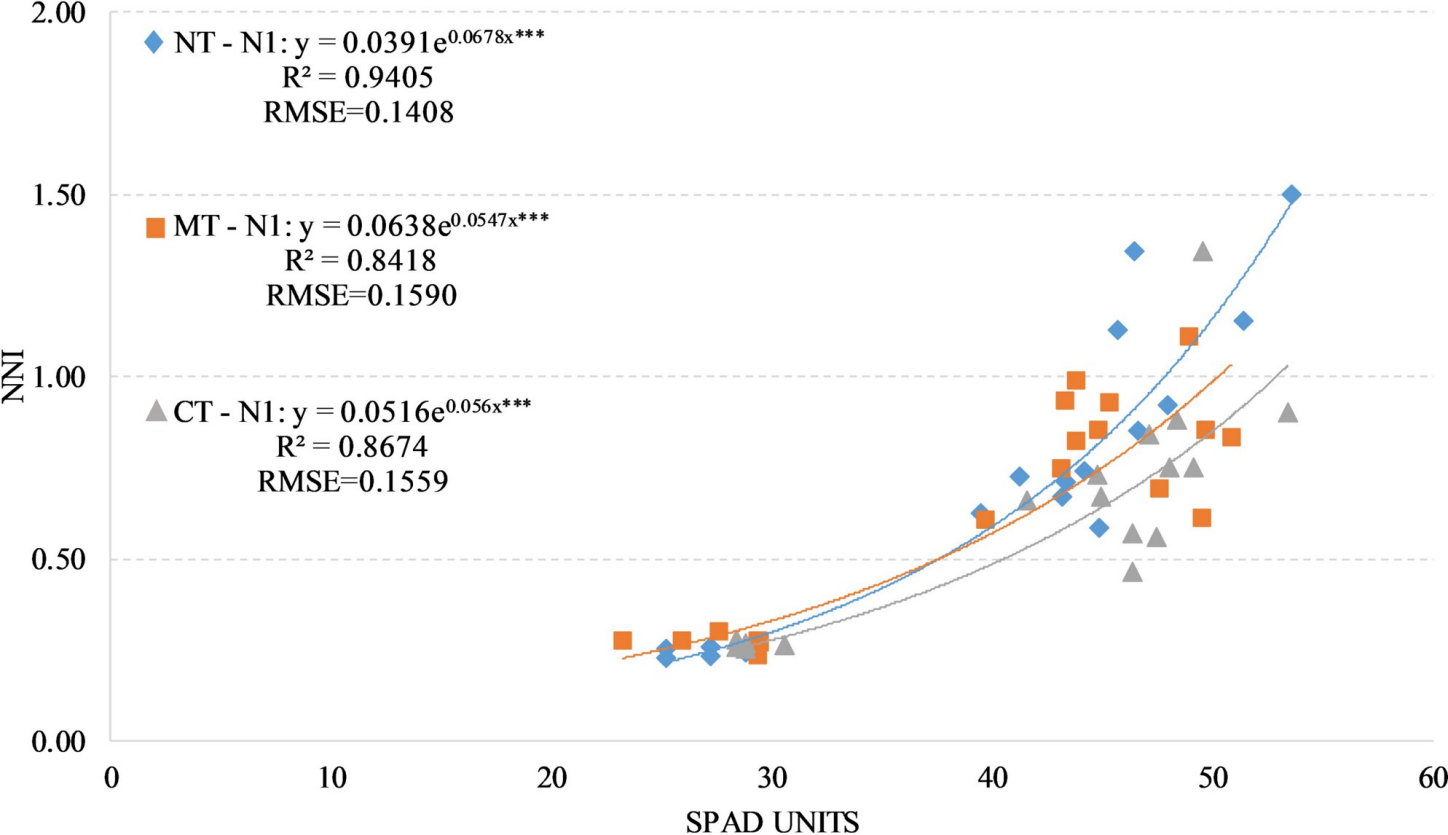
Relationship between Nitrogen Nutrition Index (NNI) and SPAD readings in durum wheat during the season 2017–2018 under N1 condition (N1, 90 kg N ha-1). NT, no tillage; RT, reduced tillage; CT, conventional tillage. ***: Significant at P level = 0.1%, RMSE, root mean square error.

**Fig 7:**
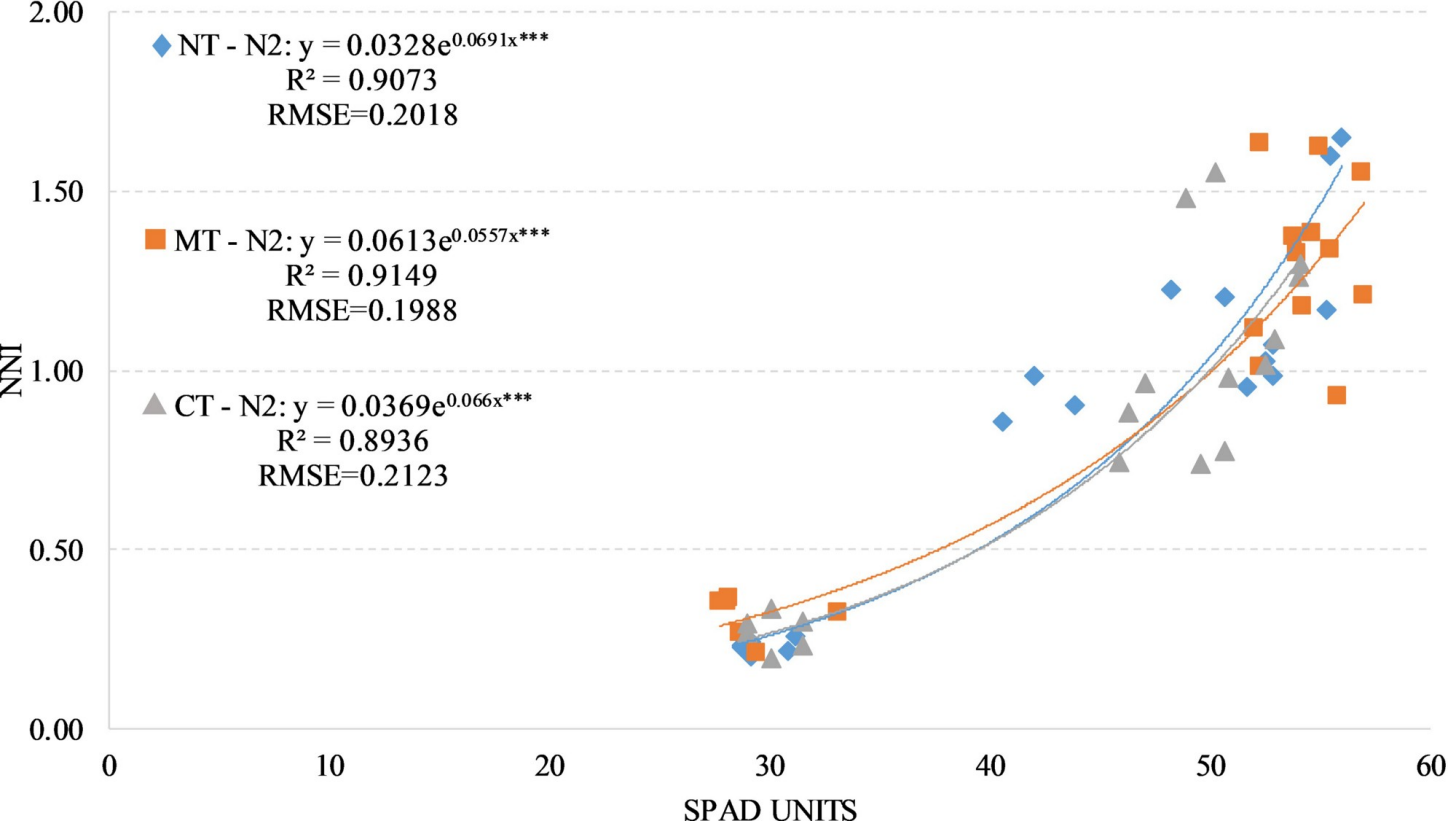
Relationship between Nitrogen Nutrition Index (NNI) and SPAD readings in durum wheat during the season 2017–2018 under N2 condition (N2, 180 kg N ha-1). NT, no tillage; RT, reduced tillage; CT, conventional tillage. ***: Significant at P level = 0.1%, RMSE, root mean square error.

We have found positive and significant exponential relationships between SPAD readings and NNI under the two fertilized treatments (N1 and N2) regardless of soil management with an average R^2^ values of 0.89 and an average RSME of around 0.18. The values of R^2^ change between the soils managements when taking the two fertilized treatments individually (Figs 6 and 7).

By evaluating absolute values of SPAD readings and leaves chlorophyll concentration, we did not find a significant difference between the soil management under the two fertilized treatments (Table 7). This find is confirmed also by analyzing the total biomass yield and the harvest index.

**Table 7:** Nitrogen Nutrition Index (NNI), total biomass yield and Harvest Index (HI) values observed (± dev.st.) under N1 and N2 fertilized treatments in the different soil managements on the season 2017–2018.

Soil management	Nitrogen input	NNI	Total biomass yield (g/m^2^)	HI
**NT**	N1	0.69 (±0.41) a	959.22 (±61.80) a	0.30 (±0.01) a
**RT**	N1	0.64 (±0.30) a	918.50 (±140.63) a	0.32 (±0.01) a
**CT**	N1	0.59 (±0.30) a	865.81 (±92.02) a	0.31 (±0.01) a
**Mean**		0.64 (±0.33)	914.51 (±104.78)	0.31 (±0.01)
**NT**	N2	0.83 (±0.48) a	1226.23 (±110.94) a	0.35 (±0.03) a
**RT**	N2	0.97 (±0.52) a	1287.05 (±246.15) a	0.32 (±0.02) a
**CT**	N2	0.80 (±0.45) a	1312.06 (±361.38) a	0.34 (±0.02) a
**Mean**		0.87 (±0.48)	1275.11 (±247.44)	0.33 (±0.03)

NT, no tillage; RT, reduced tillage; CT, conventional tillage; N1, 90 kg N ha^-1^; N2, 180 kg N ha^-1^.

^a^ value having a common letter are not significantly different at P level = 5%.

## Discussions

We observed a significant linear relationship between both SPAD readings (SPAD CP and DP) of the durum wheat leaves (Fig 1), as reported on *Triticum aestivum* L. [31]. This result allows us to simplify and reduce the readings to only one portion of the leaf, and it suggest us to perform an average between the two different readings, and use the average as a single data.

There is no fixed rule accepted by the scientific community to describe the relationship between SPAD readings, leaves chlorophyll concentration and NNI in durum wheat [19,29,41–42]. The close relationship between SPAD readings, leaf chlorophyll concentration and NNI, seem to be similar with the positive exponential relationship observed in winter wheat (*Triticum aestivum* L.) [31] or in coffee [43].

Under unfertilized treatment, NT showed a positive and exponential relationship between SPAD readings, leaves chlorophyll concentration and NNI, whit an R^2^ value of 0.77. Also, regarding the total biomass yield and the harvest index, the NT system showed significant effect compared to CT and RT in the unfertilized treatment as observed by [44] in a meta-analysis to assess and summarize the effects of NT on crop yields in different eco-regions of China.

This dynamic is probably due to the greater availability of nitrogen (1.68 vs 1.17 vs 0.98 g kg^-1^ of total nitrogen respectively in the 0–20 cm soil layer for NT, RT, CT) deriving from the higher rate of mineralization of soil organic matter presents in the NT system compared to RT and CT (25.5 vs 17.2 vs 12.6 g kg^-1^ of soil organic matter in the 0–20 cm soil layer respectively) (Table 2) as a consequence of the repeated no tillage adoption [45–46].

The increased availability of nitrogen that characterized the factorial combination NTxN0 has ensured a higher concentration of chlorophyll in the foliar tissues [36,47], which determine a greenness increase of the leaf [48–49]. These dynamics confirm the important role that no tillage plays in the increase of soil organic matter [50–51].

Under unfertilized treatment for RT and CT soil managements, there is no significant relationship between the SPAD readings and both destructive methods. This is probably due to the reduced level of soil organic matter [4–5,52–53] that allows a lower nitrogen availability in the CT and RT systems tillage [48–49].

The positive and significant exponential relationships between SPAD readings, leaves chlorophyll concentration and NNI in both fertilized treatments (N1 and N2) were significant on each compared soil managements. This confirms that the release of the nitrogen in the soil and the nitrogen supplied with fertilization are the key drivers for the SPAD accuracy to estimate the leaves chlorophyll concentration and NNI [31]. For both fertilized treatments (N1 and N2), as previously observed by [35] and [46], the total biomass yield and the HI didn’t show any significant difference between both compared soil managements, this confirms that the effect due to the mineralization of the organic matter that emerged in the NT-N0, is reduced by the nitrogen fertilization.

The R^2^ values don’t change much between the soil managements in both fertilized treatments (0.89 on average), this can be explained by the ability to flatten the differences on nitrogen availability in the compared soil management due to nitrogen fertilization [54].

## Conclusions

The Soil Plant Analysis Development (SPAD Minolta 502) is an excellent instrument to estimate the leaves chlorophyll concentration and Nitrogen Nutrition Index. We have given further information to potential users to make these SPAD readings feasible and compatible with their practices. We showed those readings on the distal (SPAD DP) and central portion (SPAD CP) are highly, and linear correlated, which would permit the users to restrict their measurements to one portion of the leaves.

From what reported in the literature, SPAD Minolta 502 is able to accurately estimate the leaves chlorophyll concentration and the nitrogen crop status (NNI) when the nitrogen doses have been provided to the crop, but also under unfertilized treatment when used for crop grown on soil with a relative high content of organic matter and nitrogen availability as consequence of continuous NT adoption.

So potential users of the SPAD Minolta 502, in order to be able to make accurate estimates of the nutritional status of the durum wheat, in addition to the calibration activities for environmental factors, cultivars factors and agronomic practices, shall take into account the endowment of organic matter mineralization and consequently the nitrogen availability.

## Supporting information

S1 File Data base. (XLSX)
